# Electron ionization of clusters containing the formamide molecule

**DOI:** 10.1140/epjd/s10053-021-00281-7

**Published:** 2021-10-20

**Authors:** Harvey-Andres Suarez-Moreno, Lauren Eckermann, Fabio Zappa, Eugene Arthur-Baidoo, Sylwia Ptasińska, Stephan Denifl

**Affiliations:** 1 Institut für Ionenphysik und Angewandte Physik and Center for Molecular Biosciences, Universität Innsbruck, Technikerstraße 25, 6020 Innsbruck, Austria; 2 Radiation Laboratory, University of Notre Dame, Notre Dame, IN 46556 USA; 3 Department of Chemistry and Biochemistry, University of Notre Dame, Notre Dame, IN 46556 USA; 4 Department of Physics, University of Notre Dame, Notre Dame, IN 46556 USA

## Abstract

Studies on electron interactions with formamide (FA) clusters promote scientific interest as a model system to understand phenomena relevant to astrophysical, prebiotic, and radiobiological processes. In this work, mass spectrometric detection of cationic species for both small bare and microhydrated formamide clusters was performed at an electron ionization of 70 eV. Furthermore, a comparative analysis of the cluster spectra with the literature-reported gas-phase spectra is presented and discussed, revealing different reaction channels affected by the cluster environment. This study is essential in developing our understanding of both low-energy electron phenomena in clusters that can bridge the complexity gap between gas and realistic systems and the effect of hydration on electron-induced processes.

## Introduction

The formamide molecule ($\hbox {OCHNH}_{2}$, here further denoted as FA) is the simplest amide molecule with high relevance because of the $\hbox {O}\!=\!\hbox {C}-\hbox {N}$ structure representing the amide (peptide) bond in chains of amino acids and proteins. The $\hbox {O}\!=\!\hbox {C}\!-\!\hbox {NH}_{2}$ group is also present in the nicotinamide molecule which is part of the electron carrier nicotinamide adenine dinucleotide and is considered a potential radiosensitizer [1]. FA is a precursor compound of carboxylic acids, amino acids, and sugar molecules [2] and was proposed to be involved in forming all four RNA nucleobases [3]. Therefore, FA is also considered highly relevant for investigating how the formation of life has emerged on Earth [2]. Its stability and possible decay are of fundamental interest for different physics, chemistry, and biology fields. FA has been found in interstellar space as well as in the planetary atmosphere of Titan. Due to the low vapour pressure of FA, 0.143 hPa at $30^{\circ }\hbox {C}$ [4], it was also suggested that FA bound on aerosols might be present on the icy surface of Saturn’s largest moon [5]. In interstellar medium, FA was detected in molecular clouds Sagittarius A, B, and B2(N) [5]. The deposition on icy grains offers the possibility of a sequence of chemical reactions upon radiation processing. Dawley et al. studied the products of pure FA and mixed FA/water ices, which were irradiated by either a 10 eV UV photon beam or a 1keV high-energy electron beam [6]. Interestingly, they observed an abundant yield of negatively charged $\hbox {OCN}^{-}$ and neutral CO, where the presence of water had a catalytic effect on the abundance of these product species. $\hbox {OCN}^{-}$, like $\hbox {CN}^{-}$ [7], may be particularly interesting species for astrochemistry since OCN and CN are pseudohalogens having a very high electron affinity [8, 9]. In [6], they also suggested that FA may become initially ionized by the radiation and the molecular cation subsequently dissociates upon recombination with an electron. Moreover, they also indicated that ionization of FA could lead to the formation of the $\hbox {OCNH}_{2}^{+}$ cation, which was proposed to be present in interstellar surroundings [10].

These previous studies devoted to possible reactions in space indicate that energetic particles and electromagnetic radiation may initiate gas-phase and condensed-phase chemistry. The interaction of this radiation with matter leads to the emission of low-energy secondary electrons with the most probable energy of about 9–10 eV and the maximum energy up to about 100 eV [11]. Depending on its kinetic energy, such low-energy electron may subsequently interact with a molecule by a variety of reactions such as dissociative electron ionization, dissociative electron attachment, and ion-pair formation. These reactions lead to chemical modification (i.e. cleavage of molecular bonds as well as possible molecular rearrangement and formation of new molecular bonds) of the involved molecular species. For example, the electron-induced synthesis of FA in an icy mixture of CO and $\hbox {NH}_{3}$ was proposed via electron attachment as well as electron ionization [12]. Moreover, FA and its analogues, containing the amide bond, a key biochemical structure, serve as model systems to understand the role of low-energy electrons in radiation damage to the cellular compounds [13, 14]. In addition to the importance of electron-induced reactions in nature, they also offer applications in controlled molecular synthesis and surface functionalization [15].

Several laboratory and theoretical studies were carried out with isolated FA in the gas phase to shed some light on possible reactions induced by low-energy electrons. For example, elastic electron scattering from FA was studied in Refs. [16–21] and inelastic electron scattering in [22]. Wang and Tian reported computational results for inelastic electron scattering as well [20]. Elastic electron scattering studies may also reveal the position of resonance, where a molecule temporarily captures the electron. The formation and subsequent decay of the anionic states of FA were investigated theoretically [23] as well as combined theoretical/experimental studies, including mass spectrometry [14, 24] or three-dimensional momentum imaging of fragment anions [25]. All previous studies with FA in the gas phase showed that the electron attachment process to FA is a dissociative one, i.e. it is not possible to detect the molecular anion on typical mass spectrometric timescales [14, 24]. This situation may change upon solvation, and therefore, this aspect was investigated in studies on anion formation of FA in the presence of surrounding molecules. Schermann and co-workers used Rydberg electron transfer to generate anions of FA clusters [26, 27] and observed indications that the excess electron is valence-bound only for $\hbox {FA}_{\mathrm {n}}$ clusters n $\ge$ 7. In contrast, the excess electron is weakly bound by dipolar and/or quadrupolar interaction in smaller clusters. These predictions on solvation of the electron were later examined in a photodetachment study of FA cluster anions and just partly supported [28]. However, electron attachment to FA embedded in helium droplets showed efficient stabilization of weakly bound FA cluster anions [29].

Some experimental data on isolated FA are also available for electron (impact) ionization. For example, the electron ionization mass spectrum of FA was previously reported by Gilpin [30] and is also available at the NIST database [31], together with early values for the ionization energy of FA (determined by Baldwin et al. [32]) and the appearance energy values of two cation fragments, $\hbox {CHO}^{+}$ and $\hbox {CH}_{2}\hbox {NO}^{+}$ (determined by Loudon and Webb [33]). In addition, the total electron ionization cross section of FA in the electron energy range from ionization threshold to 2keV was reported by Gupta et al. [34]. We note that a detailed photoionization study with FA was carried out by Leach et al. [35]. Using synchrotron photons in the energy range between 10 and 20 eV, they measured the mass spectrum and the photon yield curve for the molecular ion as well as seven fragment ions. The ionization energy and appearance energies were determined from the measured photon yield curves, respectively. Interestingly, the data in [35] indicated that photoionization at 20 eV led to substantially stronger dissociation than electron ionization at 70 eV, which was explained by the fact that autoionizing triplet states cannot be accessed by photon impact.

In this study, we have investigated electron ionization of small FA clusters at the electron energy of 70 eV. Clusters formed by supersonic expansion were crossed with an electron beam, and formed cations were analysed by mass spectrometry. For bare FA clusters, we observed ion yields up to the protonated hexamer. We also studied microhydration by adding $\hbox {D}_{2}\hbox {O}$ vapour to the expansion.

## Experimental

The present study utilized a homemade cluster source mounted to a commercial double-focusing sector field mass spectrometer (VG-ZAB-2SE). This combined setup was successfully employed for the first time in 2015 [36]. The double-focusing sector field mass spectrometer is built in reversed Nier–Johnson geometry. So, after a first field-free region, the ions pass a magnetic field, which seperates ions by momentum, and after a second field-free region, an electric field is used as energy analyzer. A channeltron-type secondary electron multiplier is used to detect the ion signal. The ions are formed in a standard Nier-type ion source. The used electron current is 10$\upmu$A. For electron ionization mass spectra, the electron energy is set to $\sim$ 70 eV. The formed ions were accelerated towards the mass analyser with an acceleration voltage of 6 kV.

The clusters are formed by supersonic expansion in a versatile cluster source consisting of a nozzle, connecting lines to a sample container and a flow controller to introduce argon as seeding gas (see [37] for a typical configuration of the cluster source). The sample containers are located outside the vacuum chamber in order to refill the container without breaking the high-vacuum after the nozzle. For the microhydration measurements, FA and $\hbox {D}_{2}\hbox {O}$ are placed in separate containers which are differentially heated. To achieve sufficient vapour pressure, the FA sample container is heated to $70 ^{\circ }\hbox {C}$ for the measurements with bare FA clusters and $75 ^{\circ }\hbox {C}$ for microhydrated FA. In the latter experiments, the $\hbox {D}_{2}\hbox {O}$ liquid is heated up to $35 ^{\circ }\hbox {C}$. The line to the nozzle as well as the nozzle itself is separately heated to avoid condensation of the vapour at cold spots ($100 ^{\circ }\hbox {C}$ and $86 ^{\circ }\hbox {C}$, respectively). Finally, the vapour is expanded with argon (2.5bar for the bare expansion and 2bar for the $\hbox {FA}+\hbox {D}_{2}\hbox {O}$ expansion) through the nozzle which has an opening diameter of $40-45 \upmu \mathrm{m}$.

After the nozzle, the formed clusters pass through a skimmer hole, 1 mm in diameter and placed about 10 mm from the nozzle, and enter the ion source chamber. During the measurements, the pressure in the cluster source chamber is about $4\times 10^{{-3}}$mbar causing a pressure of $2.5 \times 10^{{-5}}$mbar in the ion source. In the ion source chamber, a metal plate (beam flag) can be inserted to block the cluster beam before entering the ion source. This beam flag allows distinguishing between signal generated from the cluster beam and the background in the ion source chamber. When the beam flag blocks the cluster beam, the measured FA signals exclusively arise from the ionization of isolated FA molecules. In the data shown in this work, the background signals have been subtracted from the signals coming from a cluster beam. Possible negative numbers of ion intensities at the tail of some mass peaks in the shown mass spectra result from uncertainties in the mass calibration and finite statistics of mass peaks.

The chemical samples were purchased from Sigma-Aldrich Vienna, Austria, and were used as delivered. The stated sample purities were $\ge$ 99.5% in the case of FA and 99.5% for $\hbox {D}_{2}\hbox {O}$.

## Results and discussion

1. Electron ionization of bare FA clusters

The electron ionization mass spectrum of FA shown in the NIST database [31] indicates the molecular ion as the most abundant cation of FA formed by electron ionization. It thus exhibits pronounced stability of the molecule upon electron ionization. However, summing up the yield of fragment ions found in the mass spectrum also shows that about one of two FA molecules dissociates upon the impact with a 70 eV electron. Two almost equally abundant fragment cations at m/z 29 and 17 were reported with about 35% of the intensity of the parent ion [31]. The third most abundant fragment ion ($\sim$ 25%) was found at m/z 44, corresponding to the cation formed due to simultaneous hydrogen loss from the parent molecule upon ionization. All other fragment cations reported have minor abundances. The fragment cation at m/z 17 that forms via the amide (C-N) bond cleavage raised interest from a computational point of view, since this cation corresponds to $\hbox {NH}_{3}^{+}$ which forms by rearrangement in the ionized molecule [38]. In detail, a hydrogen-bonded $\hbox {H}_{2}$N...$\hbox {HCO}^{+}$ transition state was proposed, which leads to the formation of $\hbox {NH}_{3}^{+}$ $+$ CO by fast and irreversible proton transfer. The formation of $\hbox {NH}_{3}^{{+}}$ due to hydrogen transfer from the carbonyl site to nitrogen during the C-N bond cleavage was also observed in larger amides, i.e. N-methyl formamide and N, N-dimethyl formamide [39].

**Fig. 1:**
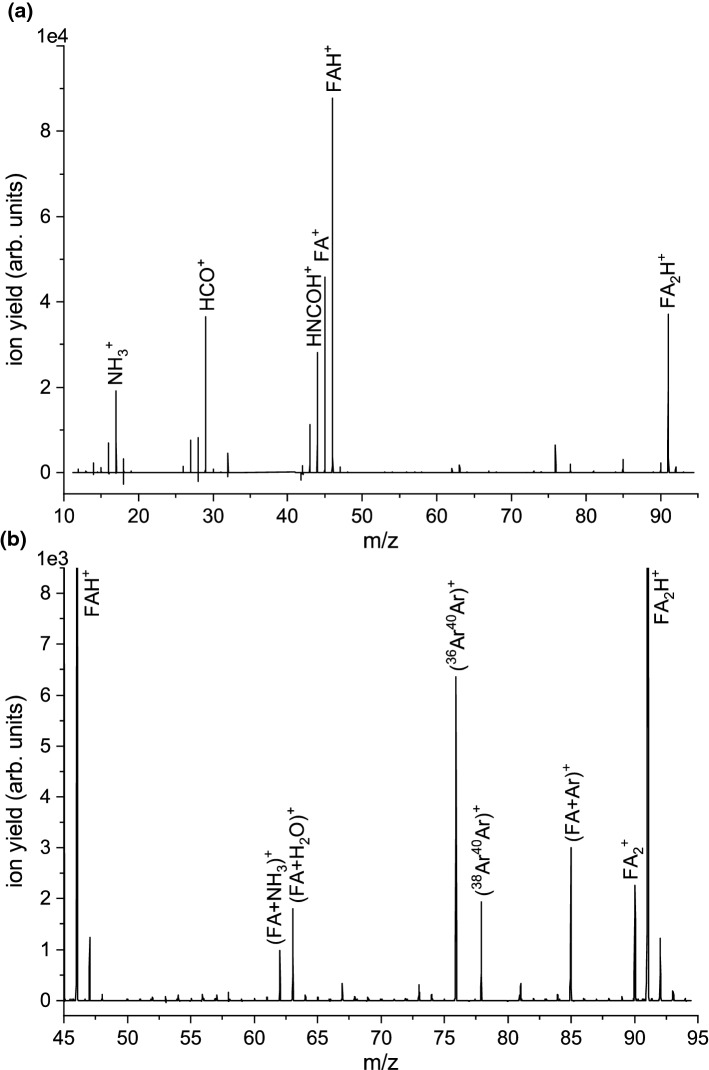
a) Lower mass section of the mass spectrum (in the range from m/z 11 to 95) with the resulting abundances of cations formed by electron ionization of bare FA clusters. The electron energy was 70 eV. The spectrum was recorded with partially closed objects slits of the mass spectrometer. Panel **b)** shows a detailed view of the ion intensities in the mass region between m/z 45 and 95. The highly intense mass peaks at m/z 40 ($^{\mathrm {40}}\hbox {Ar}^{+})$ and 80 ($^{\mathrm {40}}\hbox {Ar}_{2}^{+})$ were excluded in the measurement

**Fig. 2:**
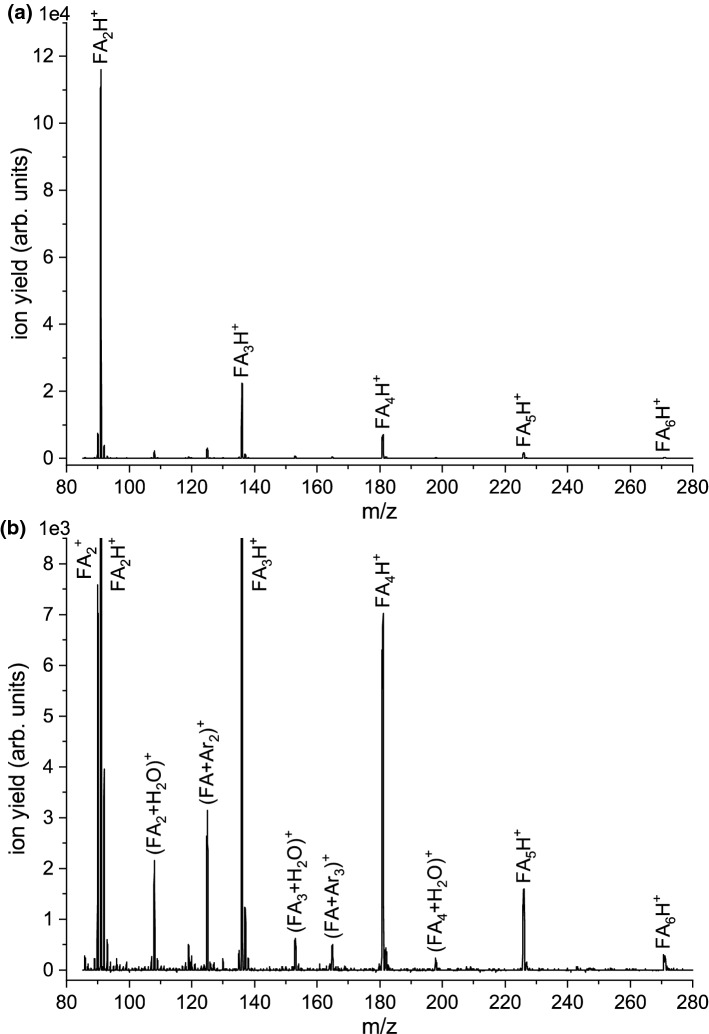
**a)** Higher mass section of the mass spectrum (in the range from m/z 85 to 280) with the resulting abundances of cations formed by electron ionization of bare FA clusters. The electron energy was 70 eV. The spectrum was recorded with fully opened objects slits of the mass spectrometer, which led to higher ion transmission through the mass spectrometer. Panel **b)** shows a detailed view of the weaker ion yields obtained in spectrum **a)**

**Fig. 3:**
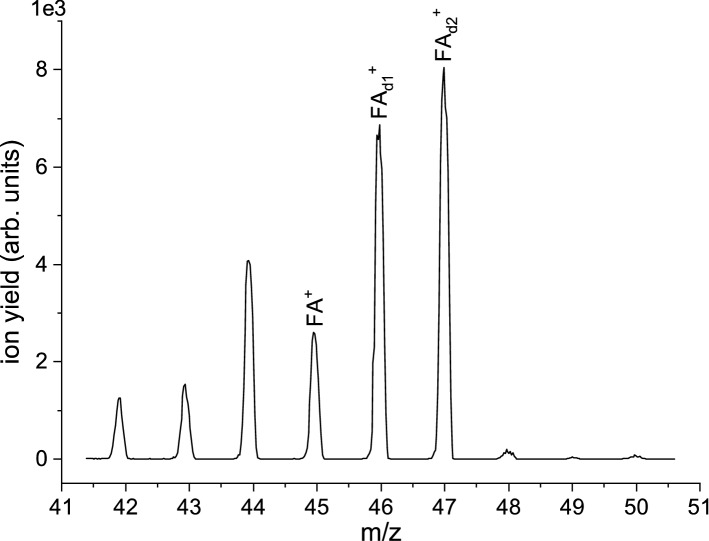
Mass spectrum (in the range from m/z 41.5 to 50.5) with the resulting abundances of cations when the cluster beam of $\hbox {FA}+\hbox {D}_{2}\hbox {O}$ was blocked by the beam flag before entering the ion source, see text. The electron energy was 70 eV

**Fig. 4:**
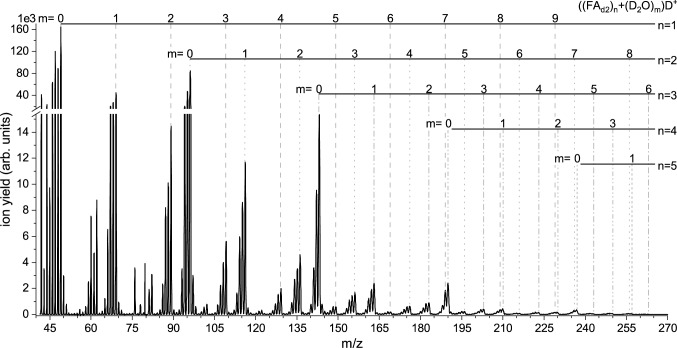
Mass spectrum (in the range from m/z 41 to 270) with the resulting abundances of cations formed by electron ionization of mixed $\hbox {FA}+\hbox {D}_{2}\hbox {O}$ clusters. The electron energy was 70 eV

**Fig. 5:**
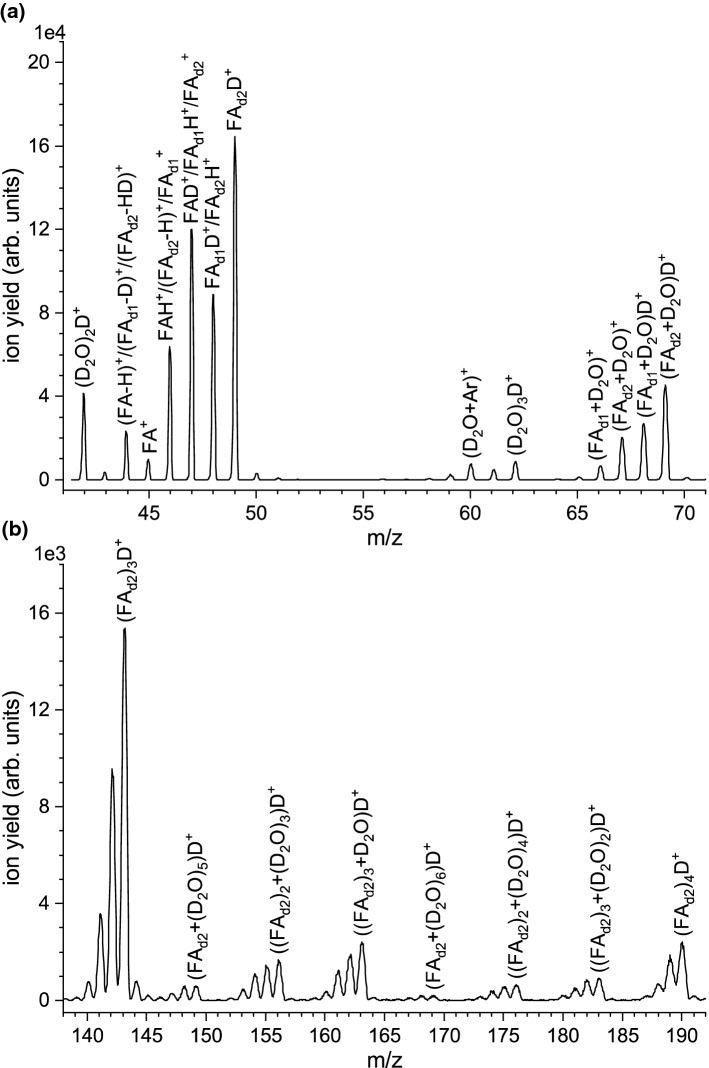
**a** Detailed view of the mass spectrum shown in Fig. 4, showing the intensity distribution in the mass range m/z 41.5–71. **b** Detailed view of the mass spectrum shown in Fig. 4, showing the intensity distribution in the mass range m/z 138–192

First, we investigated electron ionization of bare FA clusters at the electron energy of 70 eV. Figure 1a shows the resulting mass spectrum in the mass range m/z 11-95 upon electron ionization of a formamide cluster generated by the expansion with argon as seeding gas. The mass peaks at m/z 40 ($^{{40}}\hbox {Ar}^{+}$) and 80 ($^{{40}}\hbox {Ar}_{2}^{+}$) were omitted in the measurement due to exceeding ion intensity at the detector. The spectrum shown was derived by data analysis described in the experimental sections. The most abundant ionic species observed in this mass range correspond to the protonated FA monomer ion, $\hbox {FAH}^{+}$. Since the ion source chamber pressure corresponds to that of single collision conditions, $\hbox {FAH}^{+}$ is formed by the break-up of an ionized formamide cluster, most probably from the ionized dimer. The observation of protonated parent (cluster) ions is characteristic for hydrogen-containing molecules and a highly abundant species upon electron ionization of clusters [37, 40–45]. The structures and energies of the neutral formamide dimer were the focus of few theoretical and experimental studies previously. The equilibrium structure is expected to be a dimer with two hydrogen bonds (NH...O at opposite sites) between two moieties [46–48], though also a less stable isomer with just one hydrogen bond exists [49]. Detailed computational studies also exist for the protonated FA monomer, which predicted that the oxygen site is more favorable for protonation than the nitrogen site [50–52]. Possible dissociation channels of the protonated FA molecule were predicted by Nguyen et al. [52]. They proposed the formation of $\hbox {HNCOH}^{+}/\hbox {H}_{2}\hbox {NCO}^{+}$ from the ground and the first excited state of the protonated FA species. This could explain the increased abundance of the peak at m/z 44 with respect to the (unprotonated) monomer peak ($\sim$ 60%) in comparison with the mass spectrum for the isolated molecule ($\sim$ 25%). The two other prominent products at m/z 17 and 29 are also increased relative to the $\hbox {FA}^{+}$ yield, indicating that these species are also formed from clusters. However, comparing the yield of these fragment ions formed by cleavage of the C-N amide bond in relation to the total ion yield of unprotonated and protonated cluster ions shows that this bond cleavage is quenched upon ionization of FA clusters. This effect is also notable when considering the ion yields of fragment ions above the mass of the protonated monomer. Figure 1b shows a detailed view of the spectrum displayed in Fig. 1a for the mass peaks between $\hbox {FAH}^{+}$ and $\hbox {FA}_{2}\hbox {H}^{+}$. Though still detectable, the intensities of the mass peaks $(\hbox {FA}+17)^{+}$, $(\hbox {FA}+29)^{+}$ and $(\hbox {FA}+44)^{+}$, which can be associated with fragment ions of FA with one attached FA molecule, are very low compared to the ion yield of the protonated clusters and become even lower than that for weakly bonded species like $(\hbox {FA}+\hbox {Ar})^{+}$ and $(\hbox {FA}+\hbox {H}_{2}\hbox {O})^{+}$ which are probably stabilized by the evaporation of weakly bonded constituents. The clustering with water results from the presence of little residual water vapour in the cluster source. The tendency of strongly lowered abundances of molecular fragmentation (except the proton transfer reaction) continues for larger clusters series, as displayed in Fig. 2a. This figure shows the mass spectrum in an extended mass range from m/z 85 to 280, covering the largest cluster ion signal of $\hbox {FA}_{\mathrm {6}}\hbox {H}^{+}$ obtained in this study. Figure 2b shows a detailed view of mass peaks with low intensities observed in the mass spectrum plotted in Fig. 2a. Remarkably, the intensity of unprotonated molecular cluster ions $\hbox {FA}_{\mathrm {n}}^{+}$ becomes suppressed for larger cluster sizes (within the detection limit for this measurement), and only protonated clusters are formed (except for one weakly abundant peak appearing at m/z 198, which can be assigned to ($\hbox {FA}_{\mathrm {4}}+\hbox {H}_{2}\hbox {O})^{+}$).

2. Electron ionization of mixed $\hbox {FA}/\hbox {D}_{2}\hbox {O}$ clusters

In the microhydration experiment, we co-expanded $\hbox {D}_{2}\hbox {O}$ with FA and the seeding gas to study water’s effect on the electron ionization process. We used $\hbox {D}_{2}\hbox {O}$ instead of $\hbox {H}_{2}\hbox {O}$ since it may shed some light on the protonation process of the FA species. It is well known that the mass spectra of $\hbox {H}_{2}\hbox {O}/\hbox {D}_{2}\hbox {O}$ clusters are dominated by the protonated/deuteronated species [53]. Moreover, ($\hbox {H}_{2}\hbox {O})_{\mathrm {5}}\hbox {H}^{+}$ and $\hbox {FA}_{2}\hbox {H}^{+}$ have the same nominal mass, and ion signals from this pair and subsequent cluster series could not be distinguished in the recorded mass spectrum. However, though the $\hbox {D}_{2}\hbox {O}$ and the FA liquids were placed in separate containers in the cluster line, isotope exchange (most likely at the amino group of FA) leads to substantial deuteration of FA already in the cluster source before the gas expands through the nozzle. This effect is illustrated in Fig. 3 which shows the ion distribution in the mass range between m/z 41 and 51, when the flag blocks the cluster beam (flag-in). In the flag-in measurement, single FA molecules in the gas phase are ionized; therefore, one would expect isotope peaks at m/z 46 and 47 with abundances of 1.4 and 0.2 % of the intensity of the peak at m/z 45, respectively. The measurement in Fig. 3 shows abundances of about 265 and 310 instead, indicating efficient single (further denoted as $\hbox {FA}_{\mathrm {d1}})$ and double deuteration ($\hbox {FA}_{\mathrm {d2}}$) of FA in the cluster source. Figure 4 shows the resulting mass spectrum in the mass range between m/z 41 and 270 for the cluster beam of $\hbox {FA}+\hbox {D}_{2}\hbox {O}$ (corrected by the flag-in measurement). We can obtain a formamide cluster series with up to 9 $\hbox {D}_{2}\hbox {O}$ molecules attached. Notably, instead of having abundant single cluster peaks like for the spectrum for bare formamide (see Fig. 2a), a group of peaks can be obtained for each cluster size. This effect can be attributed to the presence of FA in different deuteration states. Figure 5a shows a detailed view of the intensity distribution in the mass range m/z 41-71. The intense peak at m/z 42 can be ascribed to ($\hbox {D}_{2}\hbox {O})_{2}\hbox {D}^{+}$. Comparably high intensity in this mass range is also the peak at m/z 44, which corresponds to (FA - H)$^{+}/(\hbox {FA}_{\mathrm {d1}}$ - D)$^{+}/(\hbox {FA}_{\mathrm {d2}}$ - HD)$^{+}$. Notably, m/z 44 has an increased abundance relative to m/z 45, which is weakly abundant in the cluster yield. The ratio > 1 is opposite to that for the bare FA clusters (see Fig. 1a), which may result from the situation that three isotopes (FA, $\hbox {FA}_{\mathrm {d1}}$ and $\hbox {FA}_{\mathrm {d2}})$ present in the cluster beam may contribute to m/z 44. The remaining other abundant mass peaks in the mass region of the formamide monomer are found at m/z 46–49 with slightly higher intensity. The ion yield at m/z 46 may be assigned to $\hbox {FAH}^{+}$, ($\hbox {FA}_{\mathrm {d2}}$ - H)$^{+}$ and/$\hbox {or} \,\hbox {FA}_{\mathrm {d1}}^{+}$. The peak at m/z 47 has almost factor 1.9 higher intensity than m/z 46 and represents the second most abundant peak of this group. Possible assignments of this peak are (FAD)$^{+}$, ($\hbox {FA}_{\mathrm {d1}}\hbox {H})^{+}$ and $\hbox {FA}_{\mathrm {d2}}^{+}$. One mass higher, the ion yield may be assigned to $\hbox {FA}_{\mathrm {d1}}\hbox {D}^{+}/\hbox {FA}_{\mathrm {d2}}\hbox {H}^{+}$. Only for the abundant mass peak at m/z 49, the assignment is unambiguously, and it can be assigned to $\hbox {FA}_{\mathrm {d2}}\hbox {D}^{+}$. However, due to the observed deuteration of FA in the cluster source, it is not possible to directly derive whether the deuteron was transferred from either the amino group of the partially deuterated FA species or $\hbox {D}_{2}\hbox {O}$ upon electron ionization.

In Fig. 5a, comparably weakly abundant peaks can be observed at m/z 60 and 62, which we assign to ($\hbox {D}_{2}\hbox {O}+\hbox {Ar})^{+}$ and ($\hbox {D}_{2}\hbox {O})_{3}\hbox {D}^{+}$. Electron ionization of $\hbox {H}_{2}\hbox {O}$ clusters typically yields the protonated cluster ions [54], and therefore, no significant ion yield for ($\hbox {D}_{2}\hbox {O})_{3}^{+}$ can be expected. Additional spectra at different argon pressures (not shown) also indicate that the ratio of the intensities at m/z 60 and 62 increases with higher argon pressure. At slightly higher masses (m/z 66–69), we observe ionic complexes of $\hbox {FA}_{\mathrm {d1}}$ and $\hbox {FA}_{\mathrm {d2}}$ and $\hbox {D}_{2}\hbox {O}$. In detail, we propose that the peaks can be assigned to the species ($\hbox {FA}_{\mathrm {d1}}+\hbox {D}_{2}\hbox {O})^{+}$ (m/z 66), ($\hbox {FA}_{\mathrm {d2}}+\hbox {D}_{2}\hbox {O})^{+}$, ($\hbox {FA}_{\mathrm {d1}}+\hbox {D}_{2}\hbox {O})\hbox {D}^{+}$, and ($\hbox {FA}_{\mathrm {d2}}+\hbox {D}_{2}\hbox {O})\hbox {D}^{+}$, the last one being the most abundant. The minimum-energy structures of neutral $\hbox {FA}+\hbox {H}_{2}\hbox {O}$ (and $\hbox {FA}+(\hbox {H}_{2}\hbox {O})_{2})$ complexes were recently calculated by Wang et al. [55]. They proposed that the most favourable structure for the mixed dimer has a $\hbox {C}=\hbox {O}$... H and NH...O bonding, i.e. a similar configuration with two hydrogen bonds as proposed for the FA dimer [46–48]. Maeda et al. computationally studied the structures of the ionized $\hbox {FA}+\hbox {H}_{2}\hbox {O}$ dimer [56]. They proposed migration of the water molecule around the FA moiety, where the water moves the proton from the carbon site to the oxygen site. The most stable structure has just one C-O-H... O hydrogen bond [56].

Comparing the species for the mixed clusters in Figs. 1b and 2b (as mentioned above, in the bare expansion $\hbox {H}_{2}\hbox {O}$ is present as residual vapour in the cluster source) and Fig. 5a, it is noteworthy that in Figs. 1 and 2, these mixed dimer ions are just formed in unprotonated form, while for the $\hbox {FA}_{\mathrm {(d)}}+\hbox {D}_{2}\hbox {O}$ expansion, the deuteronated ions dominate. The different neutral precursors may explain this difference for the observed product ions. If $\hbox {H}_{2}\hbox {O}$ vapour is just present in residual traces, the clustering of $\hbox {H}_{2}\hbox {O}$ molecules with FA will be weak, leading probably just to FA clusters with one water attached. If those become ionized without subsequent fragmentation, they will show up as ($\hbox {FA}_{\mathrm {n}}+\hbox {H}_{2}\hbox {O})^{+}$ in the mass spectrum; see Figs. 1b and 2b. In contrast, for the mass spectrum, as shown in Fig. 4, the co-expansion of FA and $\hbox {D}_{2}\hbox {O}$ leads to clusters containing several $\hbox {D}_{2}\hbox {O}$ molecules. The break-up of these clusters after the ionization will lead to the deuteronated ($\hbox {FA}_{\mathrm {d1/2}}+(\hbox {D}_{2}\hbox {O})_{\mathrm {m}})\hbox {D}^{+}$ species observed in the mass spectrum (detectable till $\hbox {m}=9$). Figure 5b illustrates the cluster intensities in the m/z range 138–192, which means from the formamide trimer to tetramer. All peak groups show a characteristic pattern with four distinguishable peaks with increasing intensity. The fourth peak of each group always includes deuteronated $\hbox {FA}_{\mathrm {d2}}$. Since the ratio of the four peaks does not significantly change with cluster size, we may assign all four peaks to deuteronated species containing different $\hbox {FA}/\hbox {FA}_{\mathrm {d1}}/\hbox {FA}_{\mathrm {d2}}$ isotopes. This hypothesis would align with the results for the bare clusters, which predominantly show the protonated FA cluster series.

## Conclusion

This study provides a detailed examination of the cationic mass spectra of bare FA and microhydrated FA clusters obtained from electron impact ionization of 70 eV. All of the significant cationic fragments from both types of clusters were carefully identified up to the cluster size of ($\hbox {FA}_{\mathrm {n}})\hbox {H}^{+}$ in which $\hbox {n}=6$ for the bare FA clusters and the size of ($\hbox {FA}_{\mathrm {n}}+(\hbox {D}_{2}\hbox {O})_{\mathrm {m}})\hbox {D}^{+}$ up to $\hbox {n}=5$ with several (m) molecular water attached formed from the microhydrated FA clusters. In contrast to a gas-phase FA mass spectrum in which the molecular ion is the most abundant cation formed, the mass spectra for bare FA clusters indicated that the protonated FA clusters formed from larger clusters are stable products upon electron ionization. Interestingly, the proton transfer reaction from the carbonyl to the amino group during amide bond cleavage and leading to the formation of $\hbox {NH}_{3}^{+}$, observed also in the gas phase, appears to be well pronounced for the FA clusters. In addition, another small ionic species produced, from hydrogen loss, appeared more abundant than in the gas phase. Moreover, these small fragments can be formed from the larger FA clusters. However, their total ion yield compared to the total ion yield of unprotonated and protonated cluster ions shows that the fragmentation is quenched upon ionization of FA clusters. For the microhydrated FA clusters, the mass spectra are more complex, which indicates that more reaction channels are involved due to the presence of water. However, the spectra showed a repetitive and characteristic pattern with well-identified species around clusters of a specific size. Moreover, the adventitious $\hbox {H}_{2}\hbox {O}$ in the bare FA clusters showed that only one water molecule could be attached to $\hbox {FA}_{\mathrm {n}}^{+}$, whereas the FA co-expansion with $\hbox {D}_{2}\hbox {O}$ produced clusters with several protonated and unprotonated water molecules. This observation strongly suggests the existence of different molecular precursors for both types of clusters and further supports the importance of cluster studies for understanding the effect of the environment, particularly hydration, on electron-induced processes. Overall, this study provides the initial information on ionization and fragmentation behaviour of formamide that is broadly investigated as a model for the realistic systems in astrophysics and radiobiology fields.

## Data Availability

This manuscript has associated data in a data repository. [Authors’ comment: Data shown in this work are available upon request to the corresponding author.]
